# Some carcass and meat quality characteristics of Anatolian local and Mast geese in semi-intensive conditions

**DOI:** 10.1007/s11250-026-05076-9

**Published:** 2026-05-20

**Authors:** Hasan Ürer, İskender Yıldırım, Gamze Asker, Günnur Peşmen

**Affiliations:** 1 Mucur Directorate of Agriculture and Forestry, Kırşehir, Türkiye; 2 Department of Animal Science, Faculty of Agriculture, Selçuk University, Konya, Türkiye; 3 Department of Laboratory and Veterinary Health, Suhut Vocational School, Afyon Kocatepe University, Afyonkarahisar, Türkiye

**Keywords:** Goose meat, Carcass characteristics, Meat quality, Color parameters

## Abstract

In this study, meat quality characteristics of Anatolian local geese (*Anser anser domesticus*) and Mast geese (*Anser anser*) reared under semi-intensive conditions were compared. A total of 100 geese were used: 50 from each breed, 25 males and 25 females, aged 16–18 weeks. No statistically significant difference was found between the two breeds in terms of live weight (LW) and cold carcass weight (CW) (*P* > 0.05). However, it was determined that males in both breeds had higher live weight and cold carcass weight than females (*P* < 0.05). Liver weight of Mast geese (71.81 g) was significantly higher than that of Anatolian local geese (32.91 g) (*P* < 0.05). In color analysis, the b* value (yellowness) of Anatolian local geese minced meat was higher than that of Mast geese (*P* < 0.05). For breast meat, Mast geese had a higher a* value (redness), while Anatolian local geese had a higher b* value (*P* < 0.05). No statistically significant difference was observed between breeds in terms of chemical composition (dry matter, moisture, protein, fat, ash) and physicochemical traits (pH, water holding capacity, cooking loss, and penetrometer values) (*P* > 0.05).

## Introduction

The livestock sector plays an important and ongoing role in feeding societies (Karakaya and İnci 2014), and animal proteins are essential for a healthy life (Akın and Çelen 2020). Consumers seek high-quality meat for human health. Goose meat is known as a meat with specific aroma, high-calorie, and flavour traits and compared to other meats from poultry and other animal species (Razmaite et al. 2022.) The high energy value stems from the relatively fatty nature of goose meat (Selçuk et al. 1983). Among various alternative poultry species, geese possess biological characteristics such as high growth rate, good adaptation to free movement and grazing, disease resistance, and high meat quality (Hamadani et al. 2013). The geese population in Turkey, which is expected to reach 1.303 million by 2024, constituted approximately 0.4% of the poultry population (TUIK 2024). Global production of goose meat has grown considerably over recent decades, primarily fueled by increasing demand in Asia and niche markets across Europe. However, despite its strong nutritional benefits, goose meat remains underutilized in many parts of the world. While it is highly regarded for its health benefits, its relatively high cost and the limited availability of convenient product formats hinder wider consumption. Today, consumer choices are increasingly influenced by a mix of intrinsic factors such as flavor and fat content, as well as extrinsic factors like labeling and adherence to welfare standards. Nonetheless, the lack of convenience remains a significant obstacle to making goose meat a regular dietary choice (Krunt and Zita 2026). Adequately utilizing the existing goose potential will significantly contribute to the commercial importance of goose meat for the national economy (Peşmen and Yönetken 2020). Therefore, determining the quality traits of goose meat is crucial in this regard. As organic livestock farming gains increasing importance, additional scientific research is required to ensure its sustainability and to adequately assess the current potential of goose production (Peşmen and Yönetken 2020).

A study conducted on white and spotted geese found no difference in carcass yields, and the phenotypic color variation between the varieties was reflected in the breast meat and skin (except for the redness index) (Yakan et al. 2012). Kırmızıbayrak et al. (2011) determined that age had no significant effect on skin color and meat quality traits in geese. However they significantly influenced the final breast muscle pH and redness coordinate value. The same study found that the final breast muscle pH was higher in female geese than in male geese, and the breast meat redness level was higher in breast meat obtained from male geese (Kırmızıbayrak et al. 2011). Kırmızıbayrak and Boğa (2018) obtained higher liver, foot, heart, and gizzard weights in male geese. Poultry meat quality results from complex interactions between the animal’s genotype and its environment (Le Bihan-Duval 2004). Different carcass compositions also exist among various goose genotypes (İşgüzar and Pingel 2003). The slaughter weight and carcass weight of hybrid geese are higher than those of domestic goose breeds (Kapkowska et al. 2011).

Regarding the physical properties of meat, final pH is of great importance in assessing meat quality because it can directly influence other quality traits such as meat color parameters (Kırmızıbayrak et al. 2011). In terms of the nutritional value of meat, goose meat is characterized by relatively high levels of unsaturated fatty acids and low cholesterol, as well as a low intramuscular fat content (Buzala et al. 2014). İşgüzar and Pingel (2003) reported different protein and fat contents in various Turkish goose breeds, and Wężyk et al. (2003) reported significant differences in ash content among hybrid goose breeds.

Sensory perception of meat is generally modulated by its physicochemical properties (Krunt and Zita 2026), and the sensory properties of meat are important factors determining consumer preferences for a product (Uhlirova et al. 2018). While pH, color, water holding capacity (WHC), leakage, cooking losses, and texture are important for producers for shelf life and economic reasons, consumers are more concerned with the color, texture, and sensory properties of meat. When cooking and eating poultry products, consumers often associate texture and flavor with quality. The chemical composition of meat determines its quality, and protein, fat, ash, and water are its major components (Yetişir et al. 2008). This study was conducted to determine the effects of genotype and sex on carcass and meat quality traits in Anatolian local geese (Anser anser domesticus) and Mast (Anser anser) geese raised under semi-intensive conditions. The primary hypothesis of the study is that there are significant differences in carcass composition and meat quality parameters between the two different genotypes.

## Materials and methods

### Ethics statement

Criteria specified by European policy for the protection of animals (Directive 2010/63/EU) were followed during the experimental period.

### Animal material and experimental conditions

The study involved a total of 100 geese, comprising 50 Anatolian local geese (*Anser anser domesticus*) and 50 Mast geese (*Anser anser*), with each genotype including 25 males and 25 females aged 16–18 weeks. The study was conducted on a commercial farm between July and January. Prior to slaughter, the geese were reared for two months under a semi-intensive production system.

During the daytime, birds grazed on natural pasture composed of mixed meadow vegetation. In addition to grazing, geese were supplemented with feed once daily in the evening according to the nutrient requirements for the growing period (after 4 weeks), as recommended by NRC (1994), providing approximately 15% crude protein, 3,000 kcal ME/kg diet, 0.85% lysine, 0.50% methionine + cystine, 0.60% calcium, and 0.30% available phosphorus. Clean drinking water was available *ad libitum* throughout the experimental period.

### Slaughtering and sampling

Birds were subjected to a 12 h fasting period before slaughter, with free access to water. Slaughter was carried out using standard commercial procedures. Following bleeding, carcasses were defeathered and eviscerated, and the head, feet, and visceral organs were removed. (Buckland and Guy 2002). The eviscerated carcasses were weighed, shock-frozen, and stored at − 20 °C. They were then transferred to the Meat Analysis Laboratory of the Department of Food Engineering at Selçuk University for further analyses. Eight birds representing both sexes were randomly selected from each group for chemical and physical analyses.

### Analysis methods

Chemical analyses of the goose breast meat samples (dry matter, crude protein, crude fat, and crude ash) were performed according to AOAC (2000) standards. pH (Gökalp et al. 2001), WHC (Wardlaw et al. 1973), color analysis (Hunt et al. 1991), cooking loss (CL) (Kondaiah et al. 1985), and penetrometer value (PV) (ASTM D1321) were measured. Color analyses were conducted according to the CIELab system, and results were expressed as L, a, and b* values. For meat quality analyses, samples were obtained from the breast muscle (pectoralis major). The excised breast muscle was trimmed of visible fat and connective tissue and homogenized for chemical analyses; the term “minced meat” refers exclusively to this homogenized breast muscle tissue. Meat color, WHC, CL, and tenderness measurements were performed on breast muscle samples obtained from both male and female birds.

### Statistical analysis

Statistical analyses were performed using the Minitab 19 software package (Minitab LLC, State College, PA, USA). A two-way analysis of variance (ANOVA) was applied to evaluate the effects of breed, sex, and their interaction on the measured traits. The statistical model used was as follows:

$$Yijk = {\rm{ }}\mu + Bi + Sj + (B \times S)ij + eijk$$

where Yijk represents the observed trait, µ is the overall mean, Bi is the fixed effect of breed, Sj is the fixed effect of sex, (B×S)ij is the interaction effect between breed and sex, and eijk is the random error term.

When significant differences were detected, Tukey’s multiple comparison test was used to separate group means. Results are presented as mean ± standard error (SE), and statistical significance was considered at *P* < 0.05.

## Results

The live weight (LW) and carcass weight (CW) results of Anatolian local and Mast geese are presented in Table 1. The effects of breed, sex, and their interaction were evaluated. While no significant differences were observed between breeds for LW and CW (*P* > 0.05), sex had a significant effect on both traits, with males showing higher LW and CW values than females (*P* < 0.05). Although not statistically significant, Mast geese exhibited higher LW (4.627 kg) and CW (3.678 kg) values compared to Anatolian local geese (4.499 kg and 3.593 kg, respectively).

**Table 1:** Live weight and cold carcass weight of Anatolian local geese and Mast geese according to sex (kg)

Group	*n*	LW (mean ± SE)	CW (mean ± SE)
Anatolian local geese (male)	25	4.74 ± 0.11	3.89 ± 0.12
Anatolian local geese (female)	25	4.25 ± 0.08	3.29 ± 0.08
**Sex effect (P)**		*	*
Mast geese (male)	25	4.95 ± 0.11	4.01 ± 0.11
Mast geese (female)	25	4.29 ± 0.08	3.35 ± 0.08
**Sex effect (P)**		*	*
Anatolian local geese	50	4.49 ± 0.08	3.59 ± 0.08
Mast geese	50	4.62 ± 0.08	3.67 ± 0.08
**Breed effect (P)**		0.26	0.47
**Breed × Sex (P)**		0.36	0.80

Values are presented as mean ± standard error (SE). * indicates significant differences between sexes (*P* < 0.05)

The results of minced meat and breast meat color analyses in Anatolian local geese and Mast geese are presented in Table 2. According to Table 2, the b* color coordinate was found to be 13.65 in minced meat and 6.42 in breast meat of Anatolian local geese, and 8.69 in minced meat and 1.83 in breast meat of Mast geese. Based on these results, the b* value was higher in Anatolian local geese than in Mast geese (*P* < 0.05). For breast meat, the a* color coordinate of Mast geese (15.82) was higher than that of Anatolian local geese (14.24) (*P* < 0.05). For minced meat, there was no significant difference between Mast (18.77) and Anatolian local geese (19.00) in terms of a* value (*P* > 0.05). There was also no significant difference between Anatolian local geese and Mast geese in terms of the L* color coordinate of minced and breast meat (*P* > 0.05). The breed × sex interaction effect on carcass weight was not significant (*P* = 0.80), indicating that the magnitude of sex-related differences was similar in Anatolian local and Mast geese. The breed × sex interaction effect on live weight was not significant (*P* = 0.36), indicating that sex-related differences in live weight were similar in Anatolian local and Mast geese.

**Table 2:** Meat quality traits of minced meat and breast meat in Anatolian local and Mast geese

Group	Minced meat (mean ± SE)			Breast meat (mean ± SE)		
	L*	a*	b*	L*	a*	b*
Mast geese	42.00 ± 1.9	18.77 ± 0.7	8.69 ± 0.4ᵇ	37.30 ± 1.3	15.82 ± 0.3ᵃ	1.83 ± 0.7ᵇ
Anatolian local geese	45.80 ± 1.3	19.00 ± 0.5	13.65 ± 0.3ᵃ	40.54 ± 1.2	14.24 ± 0.5ᵇ	6.42 ± 0.3ᵃ
**P value**	0.15	0.78	*	0.11	*	*

Values are presented as mean ± standard error (SE). Different letters within the same column indicate significant differences (*P* < 0.05). * indicates *P* < 0.05

^a, b^: Means with different letters in the same column indicate a statistically significant difference (*P* < 0.05). Data are presented as mean ± standard error (SE)

In the conducted meat quality analyses, Mast geese had DM 31.90%, moisture 68.10%, protein 20.07%, fat 10.05%, ash 0.95%, pH 6.02, WHC 88.37%, CL 24.84%, and PV 584.70 kg/cm², while Anatolian local geese had DM 33.24%, moisture 66.76%, protein 20.20%, fat 11.05%, ash 0.98%, pH 6.22, WHC 89.78%, CL 24.73%, and PV 594.40 kg/cm² (Table 3). No significant differences were observed between Mast and Anatolian local geese in terms of DM, moisture, protein, fat, ash, pH, WHC, CL, and PV (Table 3).

**Table 3:** Chemical composition and meat quality traits of Anatolian local and Mast geese

Group	DM (%)	Moisture (%)	Protein (%)	Fat (%)	Ash (%)	pH	WHC (%)	CL (%)	PV (kg/cm²)
Mast geese	31.90 ± 0.6	68.10 ± 0.6	20.07 ± 0.4	10.05 ± 0.9	0.95 ± 0.1	6.02 ± 0.1	88.37 ± 0.7	24.84 ± 0.6	584.70 ± 12.5
Anatolian local geese	33.24 ± 0.6	66.76 ± 0.6	20.20 ± 0.4	11.05 ± 0.9	0.98 ± 0.1	6.22 ± 0.1	89.78 ± 0.7	24.73 ± 0.6	594.40 ± 12.5
**P value**	0.27	0.27	0.68	0.54	0.53	0.16	0.29	0.91	0.59

Values are presented as mean ± standard error (SE). Differences were considered significant at *P* < 0.05

## Discussion

This research compares carcass and meat quality traits between Anatolian local geese (*Anser anser domesticus*) and Mast geese (*Anser anser domesticus*, commercial line) raised under semi-intensive conditions to highlight differences and similarities.

No significant difference was found between the two genotypes in terms of live weight and cold carcass weight (*P* > 0.05), indicating that both genotypes exhibited similar growth performance. However, in both breeds, males were found to have higher live weight and cold carcass weight compared to females (*P* < 0.05). This finding is consistent with previous studies confirming the effect of sex on growth performance (Kırmızıbayrak et al. 2011; Yakan et al. 2012). Although only cold carcass weights were measured and presented in this study, hot carcass weights can be estimated to be approximately 2–4% higher, based on typical chilling losses reported for poultry carcasses. During the chilling process, carcasses inevitably lose moisture due to evaporation and drip loss, which results in a measurable reduction in weight. Previous studies have consistently reported chilling losses within this range for poultry, including waterfowl. Accordingly, the hot carcass weights of the geese evaluated in the present study are expected to be slightly higher than the reported cold carcass values. This consideration is important when comparing carcass yield results with studies reporting hot carcass weights and should be taken into account when interpreting differences across production systems and carcass evaluation methodologies. The absence of hot carcass weight measurement is not expected to affect the interpretation of the results, as all carcass traits were assessed under identical chilling conditions and comparisons were made within the same experimental framework.

The significantly higher liver weight of Mast geese (71.81 g) compared to Anatolian local geese (32.91 g) (*P* < 0.05) suggests that genotype may play a determining role in internal organ development. It has been reported that this difference in liver weight may result from factors such as rearing system, genotype, and slaughter age (İşgüzar and Pingel 2003; Tilki et al. 2004; Çelik and Bozkurt 2009). The fact that these values are lower than the liver weights reported by Sole et al. (2016) and Peşmen and Yönetken (2020) may be attributed to differences in rearing conditions.

Among the color parameters, the b* value (yellowness) was found to be statistically higher in Anatolian local geese in both minced and breast meat (*P* < 0.05). This indicates that Anatolian local geese meat has a more yellowish color tone. This difference in meat color may be due to the accumulation of carotenoid pigments in muscle tissue or differences in fat composition depending on the genotype (Biesek et al. 2020). The higher a* value (redness) in the breast meat of Mast geese indicates that the meat of this genotype has a redder hue. Meat color in poultry is influenced by factors such as myoglobin content and post-slaughter biochemical processes (Fletcher, 2002). Studies by Baeza et al. (1997) and Santé et al. (1991) have reported that selection for meat yield may result in paler breast meat.

In terms of chemical composition (DM, protein, fat, ash) and physical parameters (pH, WHC, CL, PV), no statistically significant differences were observed between the breeds (*P* > 0.05). This suggests that both genotypes have similar technological meat quality and nutrient composition. Similarly, Yakan et al. (2012) reported no significant differences in chemical composition among different goose genotypes. Çelen and Güngördü (2009) also reported no differences in breast meat moisture, protein, and fat content among broiler chicken genotypes.

The similarity in WHC and CL values between the two breeds indicates that genotype has a limited effect on the meat’s water retention properties. Işık (2008) reported that WHC may vary among chicken genotypes, while Jassim et al. (2011) noted that in ducks, WHC can significantly differ depending on breed, age, and carcass parts. The lack of a statistically significant difference in PV suggests that the two breeds have similar meat texture. Işık and Yetişir (2010) reported average PV of 530 kg/cm² in different chicken genotypes, and Santos et al. (2004) found that genotype had an insignificant effect on chicken breast meat firmness.

This study demonstrated that, under semi-intensive conditions, the local and Mast goose breeds exhibited general similarities in terms of carcass weight, chemical composition, and physicochemical properties. However, statistically significant genotype differences were observed in color parameters (L, a, b*) and liver weight. Additionally, in both breeds, sex was found to have a significant effect on live weight and carcass weight. The findings indicate that breed and sex selection in goose production may influence certain carcass and meat quality traits.

The absence of a significant breed × sex interaction for carcass weight (*P* = 0.80) indicates that the magnitude of sex-related differences were similar in Anatolian local and Mast geese. This finding suggests that the effect of sex on carcass weight is largely independent of breed and may be governed by comparable physiological and growth-related mechanisms across genotypes. Although males exhibited higher carcass weights than females, the consistency of this pattern between breeds implies that sex represents the primary determinant of carcass weight, rather than breed-specific responses. The lack of a significant breed × sex interaction for live weight (*P* = 0.36) indicates that the effect of sex on growth performance was consistent between Anatolian local and Mast geese, suggesting similar growth patterns across breeds.

## Data Availability

The contents underlying the research text are included in the manuscript.
